# Supplementary material for: Two-stage opening of the Dover Strait and the origin of island Britain
Source: Nat Commun. 2017 Apr 4;8:15101. doi: 10.1038/ncomms15101 (PMC5382280; doi:10.1038/ncomms15101)
Supplement: Supplementary Information — Supplementary Figures and Supplementary Tables [file ncomms15101-s1.pdf]

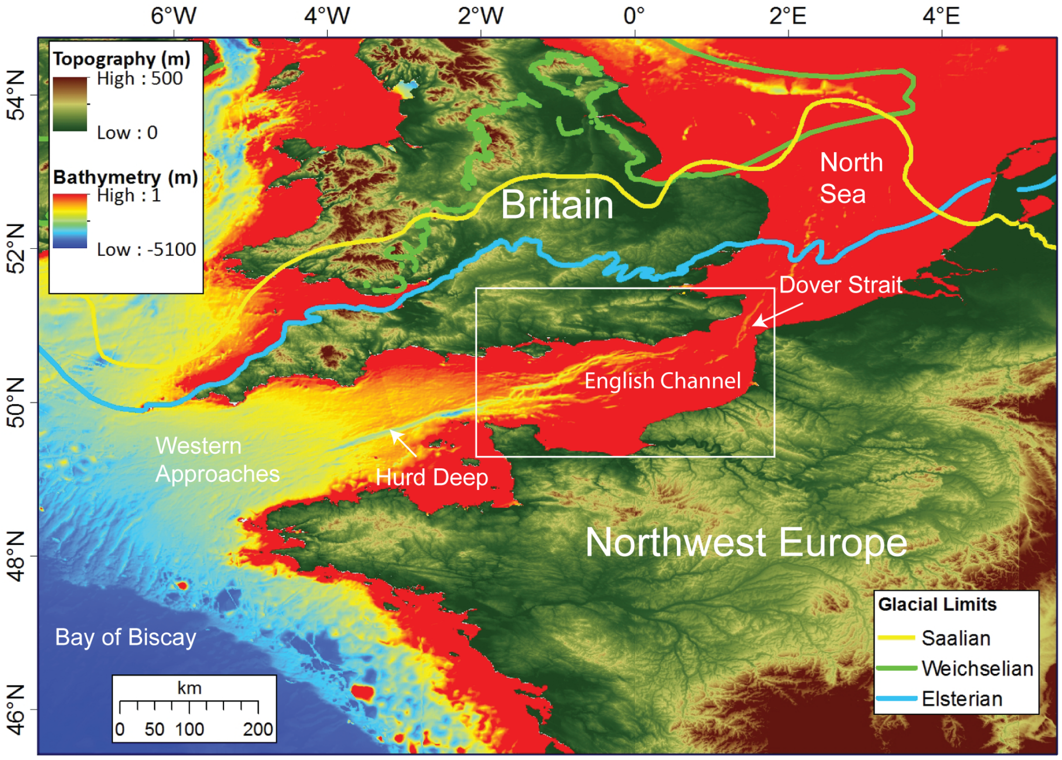

Supplementary Figure 1. Topographic map of the English Channel region plotted with a WGS-1984 projection. The Quaternary glacial limits are from Ehlers et al[1]. White box indicates areal extent of Figure 1. Onshore elevation is from SRTM[2] and offshore bathymetry is from GEBCO[3].

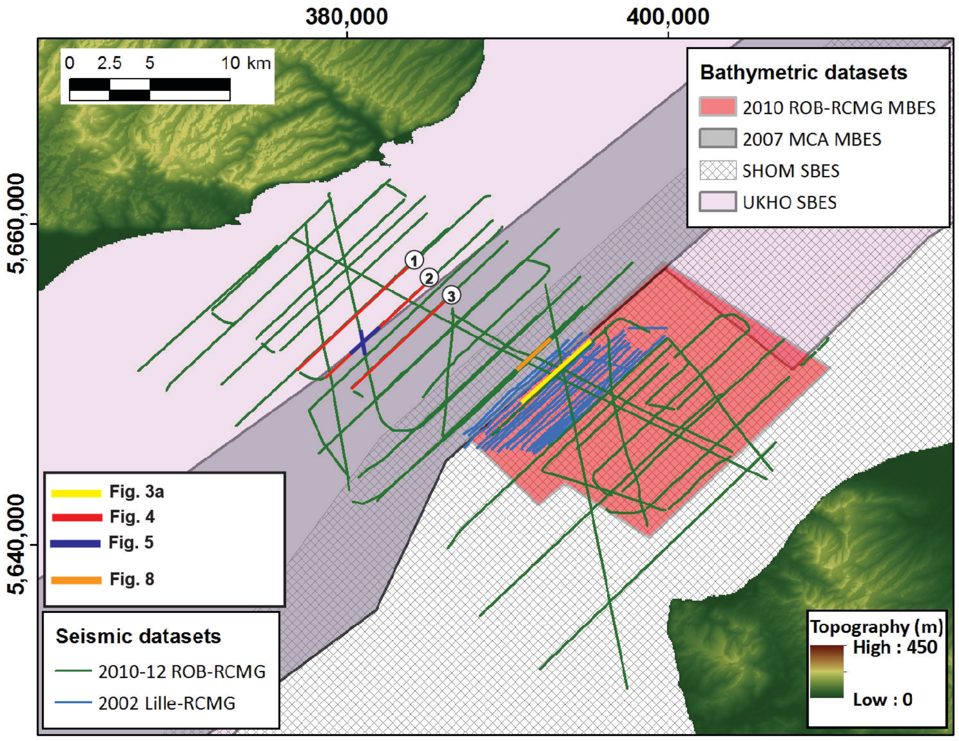

Supplementary Figure 2. Map of the Dover Strait showing locations of seismic and digital bathymetric datasets used in this study. The locations of seismic lines used in Figures 3a, 4, 5 and 8 are indicated.

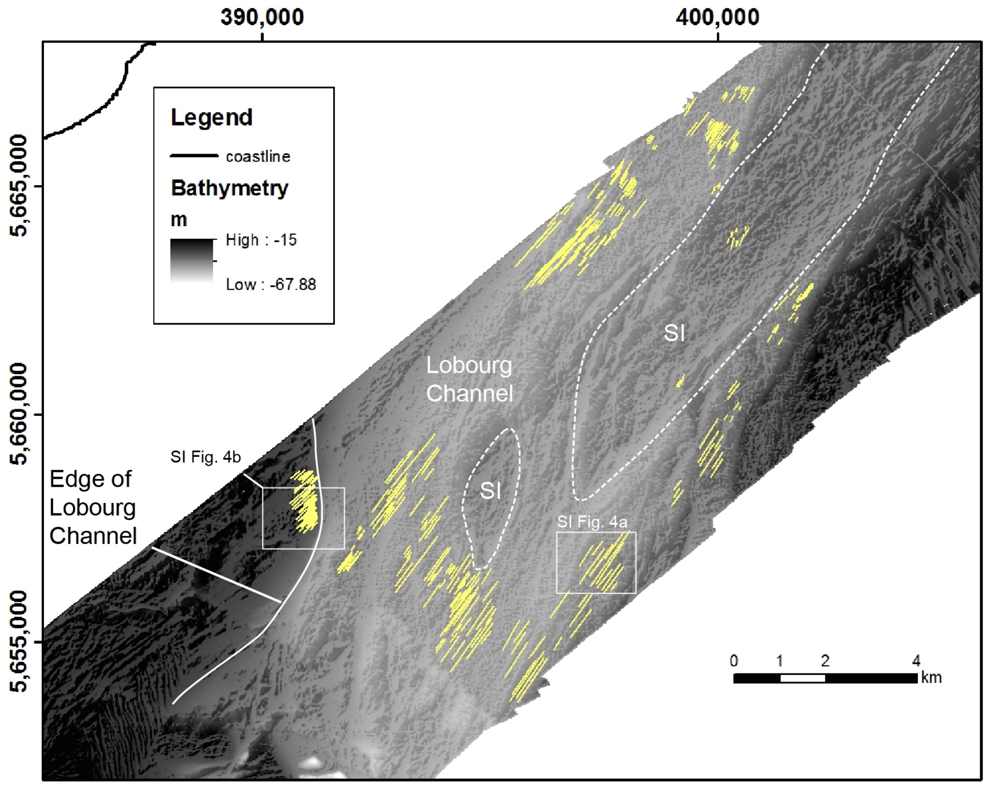

Supplementary Figure 3. Map showing distribution and orientation of longitudinal lineations identified in multibeam bathymetric data from the Lobourg Channel. Longitudinal lineations are indicated in yellow. The background image is grey-scale multibeam bathymetry image. SI, streamlined island. Locations of Supplementary Figs 4a and 4b are indicated. Note one set of lineations is eroded on north-west flank of Lobourg Channel (Supplementary Fig. 4b).

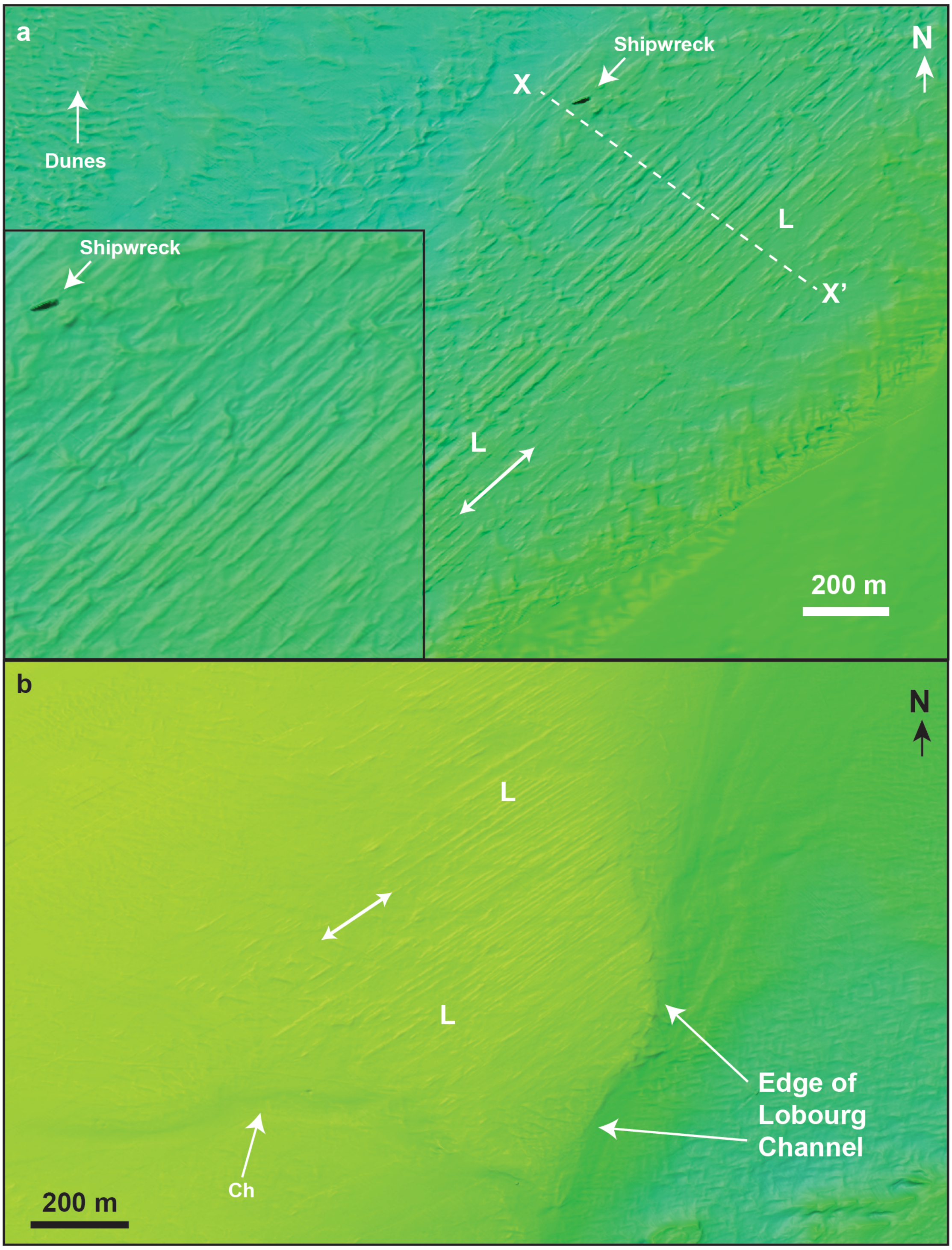

Supplementary Figure 4. Multibeam bathymetry images showing details of longitudinal lineations (L) eroded into Chalk bedrock. The orientation of lineations is shown by double-headed arrows. Location of images is indicated in Fig. 7a and Supplementary Fig. 3. Water depth as in colour bar in Fig. 7a. **a,** Lineations at SE margin of Lobourg Channel. Inset shows detail of bedforms. X-X’ shows line of topographic section in Supplementary Fig. 5. **b,** Lineations eroded into north-west flank of Lobourg Channel. Ch, small channel.

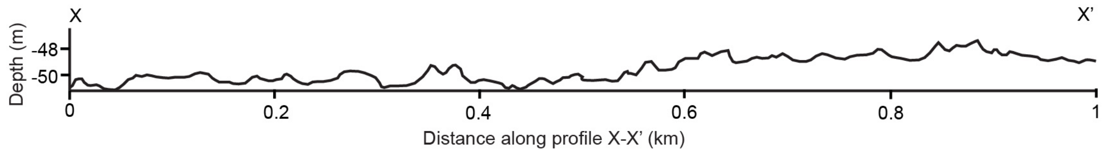

Supplementary Figure 5. Topographic profile perpendicular to elongation direction of longitudinal lineations. Line of cross-section shown in Supplementary Fig. 4a.

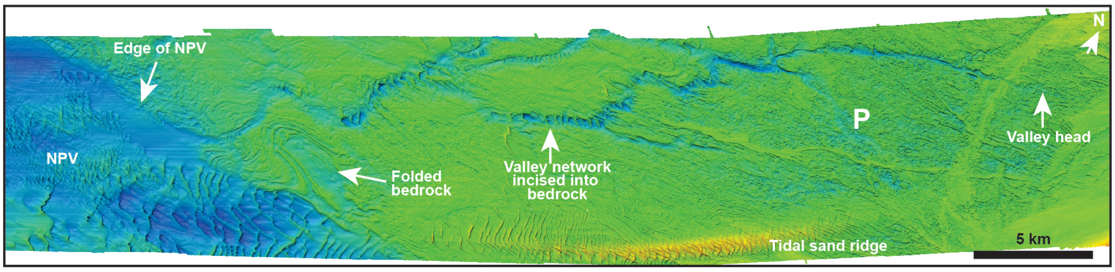

Supplementary Figure 6. Multibeam bathymetry image showing detail of prominent valley eroded into bedrock of Platform P. Note how valley network heads on platform indicating that it is not connected to onshore drainage. NPV, Northern Palaeovalley.

Supplementary References

1 Ehlers, J., Gibbard, P. L. & Hughes, P. D. *Quaternary glaciations-extent and chronology: a closer look*. (Elsevier, 2011).

2 Farr, T. G. *et al.* The Shuttle Radar Topography Mission. *Reviews of Geophysics* **45**, doi:10.1029/2005rg000183 (2007).

3 GEBCO. *General Bathymetric Chart of the Oceans - GEBCO_08 Grid, version 20081212.* www.gebco.net, 2008).
